# The effectiveness of tenofovir-based pre-exposure prophylaxis for prevention of HIV acquisition among sub-Saharan African women at high risk: a systematic review

**DOI:** 10.11604/pamj.2021.38.308.26014

**Published:** 2021-03-26

**Authors:** Grant Murewanhema, Moffat Malisheni, Noah Fongwen Takah

**Affiliations:** 1 Department of Obstetrics and Gynaecology, College of Health Sciences, University of Zimbabwe, Avondale, Harare, Zimbabwe,; 2 Ministry of Health, Lusaka, Zambia,; 3 Limbe Regional Hospital, Ministry of Public Health, Yaoundé, Cameroon,; 4 International Diagnostics Centre Africa, Addis Ababa, Ethiopia

**Keywords:** Human immunodeficiency virus, sub-Saharan Africa, pre-exposure prophylaxis, tenofovir, vaginal gel, truvada, effectiveness

## Abstract

**Introduction:**

women in sub-Saharan Africa (SSA) are disproportionately affected by the HIV epidemic. In 2019, they constituted 59% of new infections; thus, they remain a key population for control. Public health interventions to prevent acquisition of HIV in this high-risk population are urgently needed. Tenofovir-based pre-exposure prophylaxis (TFV-PrEP) has been shown to reduce HIV infections in other key populations. However, comprehensive evidence regarding TFV-PrEP effectiveness in women living in SSA has not been determined. Therefore, we undertook a systematic review to determine the effectiveness of tenofovir-1% (TFV-1%) vaginal gel, oral tenofovir (TFV) and tenofovir-emtricitabine (TDF-FTC) pre-exposure prophylaxis for primary acquisition of HIV in at-risk women living in SSA.

**Methods:**

OVID Medline, Embase, CENTRAL, Web of Science and Clinical Trials.gov were searched for eligible studies from 1^st^ January 2020 to 31^st^ July 2020. Only randomised controlled trials (RCTs) conducted in women living in SSA were included. Measures of effectiveness (hazard ratios (HR), incidence rate ratios (IRR)) were extracted from individual studies to determine the effectiveness of TFV-PrEP in preventing HIV infection among at-risk women living in SSA.

**Results:**

from 2002 non-duplicate articles, four RCTs evaluating the effectiveness of one or more of the interventions against placebos were included. TFV-1% vaginal gel, oral TDF or TDF-FTC were not effective in preventing the acquisition of HIV infection in women living in SSA. However, poor adherence by study participants could have confounded the true effectiveness of TFV-PrEP in this high risk population. Meta-analysis was not conducted given the limited number of eligible studies identified from the search.

**Conclusion:**

the current evidence does not support the effectiveness of TFV-PrEP for HIV in SSA women. More studies aimed at addressing factors driving low adherence to HIV interventions in this high risk population are urgently needed in order to improve the design of future RCTs leading to the determination of more reliable estimates of TFV-1% vaginal gel or oral TDF or TDF-FTC effectiveness. Protocol registration: this systematic review was not registered in PROSPERO.

## Introduction

Recent UNAIDS estimates show that in sub-Saharan Africa (SSA), women and girls constituted an estimated 59% of new HIV infections in 2019 [1]. Despite a marked reduction in incident HIV infections by 40% since the peak in 1998, SSA women remain at substantial risk of acquiring HIV [1]. Young women remain a driving factor for the HIV epidemic and are a key population for control [2,3].

Pre-exposure prophylaxis (PrEP) refers to use of antiretroviral drugs by an individual at risk of HIV acquisition to prevent infection before the exposure occurs. The World Health Organisation (WHO) has included oral tenofovir-containing drugs for use as PrEP in groups at substantial risk of HIV acquisition [4]. Efficacy of tenofovir-based pre-exposure prophylaxis (TFV-PrEP) was demonstrated in studies among men who have sex with men (MSM) and transgender women (TGW), with a 44% reduction (95% CI 15-63%, p=0.005) in HIV incidence attributable to Truvada (TDF-FTC) [5].

Women in the patriarchal African society usually have little or no control over the preventive methods available [6] and the search for effective pharmacological interventions continues to complement behavioural interventions [7]. Based on results from studies conducted in MSM and TGW [8,9], TFV-PrEP offers a viable alternative of control for women at high-risk of HIV acquisition. However, comprehensive evidence regarding TFV-PrEP effectiveness in women living in SSA has not been determined. We undertook a systematic review to determine the effectiveness of TFV-PrEP for prevention of acquiring HIV among at high-risk women residing in SSA.

## Methods

**Protocol and registration:** this systematic review was conducted following preferred reporting items for systematic reviews and meta-analysis (PRISMA) guidelines [10,11] with additional guidance derived from the cochrane handbook of systematic reviews and meta-analysis for interventions [12]. The protocol for this systematic review was not registered in PROSPERO.

**Eligibility criteria:** the studies were restricted to heterosexual SSA women at risk of HIV acquisition. No limitation was placed on language. The interventions evaluated against their respective comparator placebos were TFV-1% vaginal gel, oral TDF and oral TDF-FTC. The outcome was effectiveness against HIV acquisition. Only randomised controlled trials (RCTs) evaluating the effectiveness of the study products were included.

**Information sources and search strategy:** a systematic literature search in Medline, Embase, Web of Science, CENTRAL and ClinicalTrials.gov through OVID was conducted from 1^st^ January 2020 to 31^st^ July 2020. Multiple databases were searched to limit bias as recommended by the cochrane collaboration [12]. The search strategies were constructed from combinations of medical subject headings (MeSH) and keywords, which were adjusted for the individual databases by the principal investigator (GM) with inputs from MM and NFT. The cochrane highly sensitive strategy for identifying RCTs in Medline: sensitivity-maximising version (2008 revision); OVID format [12] was used to filter RCTs in Medline whilst the Scottish intercollegiate network search filters for RCTs [13] were utilised in Embase. No filters were applied in the remaining databases. Detailed search strategies in OVID Medline and Embase are shown in Annex 1 and Annex 2.

Results from each database were imported into Mendeley desktop version 1.19.4 and de-duplicated. GM, MM and NFT screened the titles and abstracts of the remaining studies for eligibility independently. All eligible studies were accessed except for the FACTS-001 study [14] for which only a conference abstract was found. All studies for which eligibility could not be determined based on the abstract were obtained in full for further assessment.

**Data collection process and data items:** a pre-tested data extraction sheet was developed in Microsoft Excel 2013 to facilitate data collection. Study lead author, year of publication, geographical location, source of funding, journal of publication, number of participants and duration of follow-up were extracted. Age and reported measures of central tendency and spread were also extracted. Outcome specific headings used in the effectiveness review included effectiveness of each of TFV-1% vaginal gel, oral TDF and TDF-FTC. Odds ratios (OR), relative risk (RR), hazard ratios (HR) and incidence rate ratios (IRR) were extracted as summary measures.

**Risk of bias assessments:** the cochrane collaboration tool for assessing the risk of bias in RCTs was used [12,15]. It is designed to identify selection, performance, attrition, detection, reporting and other biases.

**Statistical analysis:** the results from the RCTs evaluating effectiveness of each of the study products against placebo were synthesized. We intended to conduct pairwise meta-analysis for each of TFV-1% vaginal gel, oral TDF and TDF-FTC against respective placebos in Stata version 11. Since the studies were conducted across different geographical locations and years, a source of significant heterogeneity, random effects meta-analyses were to be conducted, with fixed effects meta-analyses as sensitivity analyses. We obtained a limited number of studies and therefore we could not conduct meta-analysis, tests of heterogeneity and meta-regression. Heterogeneity was to be assessed using the I^2^. We could also not produce funnel plots to assess risk of publication bias owing to limited number of studies.

## Results

**Figure 1:**
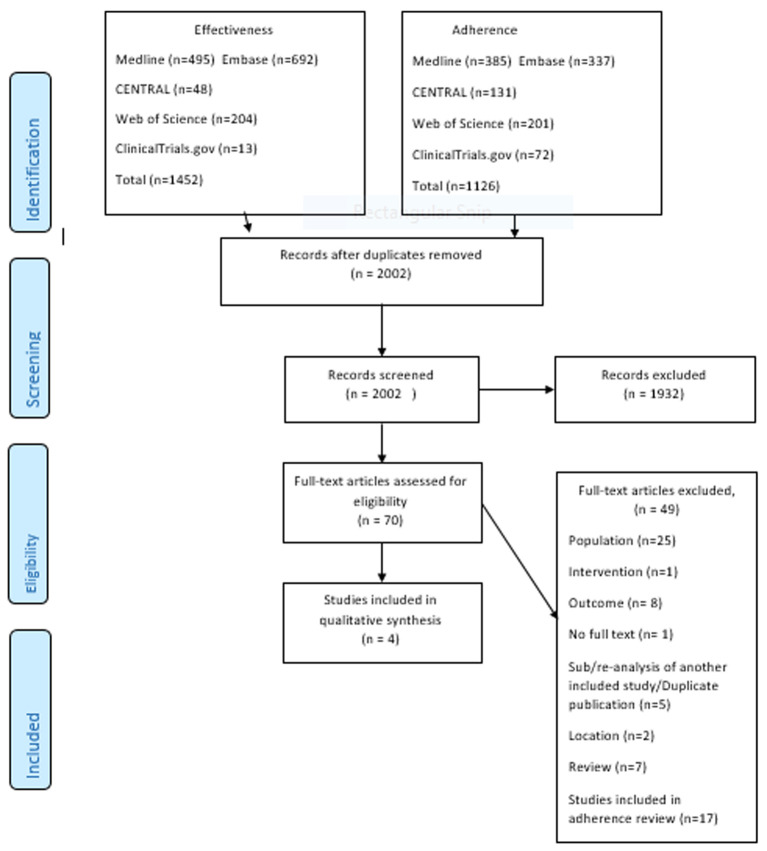
PRISMA flow diagram

Out of 2002 studies screened after removing duplicates, 70 full-text articles were assessed for eligibility and four RCTs evaluating effectiveness of interventions and were included in synthesis for effectiveness. The study selection is detailed in the PRISMA flow diagram in Figure 1.

**Characteristics of included studies:** all studies were published in English, mostly between 2014 and 2015. They were conducted across centres in South Africa, Cameroon, Ghana, Nigeria, Uganda, Zimbabwe, Kenya and Tanzania. Gilead sciences (TDF, TDF-FTC) and CONRAD (TFV-1% vaginal gel) provided the study products. Financial support was provided by the United States Agency for International Development (USAID), Family Health International 360 (FHI-360), the Centre for the AIDS Programme of Research in South Africa (CAPRISA), National Institutes for Health (NIH) and Bill and Melinda Gates Foundation. Peterson *et al*. reported significant conflicts of interests where individuals affiliated to sponsors were closely involved in study design, manuscript writing, revision and decision to publish [16]. All enrolled black SSA women, absolute ages 18-45 years with variations in the means/medians reported across the different studies. Adolescents below 18 years, who are also at risk, were excluded because of the need for parental/guardian consent. The RCTs are fully characterised in Table 1.

**Table 1:** characteristics of included studies

Author, year of publication, journal, trial name	Location	Funding source/ author declaration of financial interest	Study design/ study products	Number of participants/ follow-up	Study participants characteristics
Karim QA *et al*. 2010, Science CAPRISA-004 (and Mansoor *et al*. 2014, AIDS Behav)	2 CAPRISA Clinical Research Sites, KwaZulu-Natal, South Africa	Sponsors: USAID, FHI-360; study products: CONRAD, Gilead Sciences; competing interests: lead author was co-principal investigator for HPTN. No other competing interests declared	Design: phase 2b double-blind, placebo-controlled RCT; products: TFV-1% vaginal gel and placebo gel	889 participants (445 TFV arm, 444 placebo arm); enrolment and follow-up for 21 months from May 2007 to January 2009	Rural women: n=611; mean age 23.3 years, range 18-40 years; 6.5% married; 77% stable partner; mean number of lifetime sexual partners 2.1; urban women: n=278; mean age 25.1 years, range 18-40 years, 3.6% married, 93.1% stable partner; mean number of lifetime sexual partners 6.0
Peterson L *et al*. 2007, PLoS Clinical Trials	Research sites in Tema, Ghana; Douala, Cameroon and Ibadan, Nigeria	Sponsors: Bill and Melinda Gates Foundation and FHI-360; study product: Gilead Sciences; competing interests: one author was with the Bill and Melinda Gates Foundation and another with Gilead Sciences; both contributed to study design and manuscript writing and publication decision; another author was both an employee and shareholder of Gilead Sciences	Design: phase 2 double-blind, placebo-controlled RCT; products: TDF 300mg and oral placebo	936 participants; 469 on TDF (Ghana 200, Cameroon 200 and Nigeria 69); 467 on placebo (Ghana 200, Cameroon 200, Nigeria 67); enrolled and followed up monthly between June 2004 and March 2006	Mean age: TDF group 23.6 ± 3.9 years; placebo group 23.5 ± 3.9 years; not married: TDF group 92.7%, placebo group 89.1%
Marrazzo JM *et al*. 2015, N Eng J Med VOICE/MTN-003	15 clinical research sites in South Africa, Uganda and Zimbabwe	Sponsors: NIH study products: CONRAD and Gilead Sciences; competing interests: no conflicting financial interests were disclosed	Design: phase 2b double-blind, placebo-controlled RCT; products: TFV-1% vaginal gel, placebo gel, TDF 300mg and TDF-FTC 300mg/200mg and oral placebo	5029 participants; TDF 1007, TDF-FTC 1003, Oral placebo 1009, TFV gel 1007, placebo gel 1003; enrolled and followed-up monthly from September 2009 to June 2011	Mean age 25.3 ± 5.2 years, 21% married, 22% had ≥2 male partners in the past 3 months
Van Damme L *et al*. 2012, N Eng J Med FEM-PrEP	Clinical Research Sites in Arusha, Tanzania; Bondo, Kenya and Bloemfontein and Pretoria, South Africa	Sponsor: USAID and Bill and Melinda Gates Foundation; study product: Gilead Sciences; competing interests: no significant financial interests were declared	Design: phase 3 double-blind placebo-controlled RCT; product: TDF-FTC 300mg/200mg and oral placebo	2120 participants; (63 Arusha, 739 Bondo, 554 Bloemfontein, 764 Pretoria); TDF-FTC 1062, Placebo 1058; enrolled and followed-up monthly from June 2009 to April 2011	Mean age 24.2 years (median 23 years, range 18-35); 30.9% married; 12.6% reported sex for money or gifts with a non-primary partner in the previous 4 weeks

All enrolled HIV-negative, non-pregnant, non-lactating women. HIV testing was performed in standard laboratories using standard kits to ensure uniformity of measurement and reduce misclassification bias. Study participants underwent monthly HIV testing, adherence measurement and risk-reduction counselling. Additionally, they were provided with standard HIV prevention packages. Karim *et al*. [17], Peterson *et al*. [16] and van Damme *et al*. [18] evaluated the effectiveness of TFV-1% vaginal gel, oral TDF and TDF-FTC respectively against placebos in 1: 1 randomisation ratios whilst Marrazzo *et al*. [19] evaluated TFV-1% vaginal gel, oral TDF and TDF-FTC against placebos in a complex five-arm 1: 1: 1: 1: 1 trial. Effective randomisation was concluded by even distribution of baseline participant characteristics across all the studies; however, it was also notable that 62.8% of participants in the multi-national, multi-centre trial by Marrazzo *et al*. [19] were from Durban sites. Karim *et al*. reported differences with rural women being much younger, poorer and reported lower sexual frequency and condom use than their urban counterparts [17].

All studies followed the consolidated reporting of trials (CONSORT) 2010 guidelines and included CONSORT flow diagrams accounting for study participants [20,21]. Only Karim *et al*. [17] and the TDF-FTC arm of Marrazzo *et al*. [19] were able to follow-up participants for the originally planned follow-up period. The trial by van Damme *et al*. [18] and the TFV-1% vaginal gel and oral TDF arms of the trial by Marrazzo *et al*. [19] were stopped prematurely by data safety and monitoring bodies (DSMB) due to futility. The trial by Peterson *et al*. was stopped prematurely in Nigeria due to poor protocol compliance and in Cameroon due to post-trial care of sero-converters [16].

**Figure 2:**
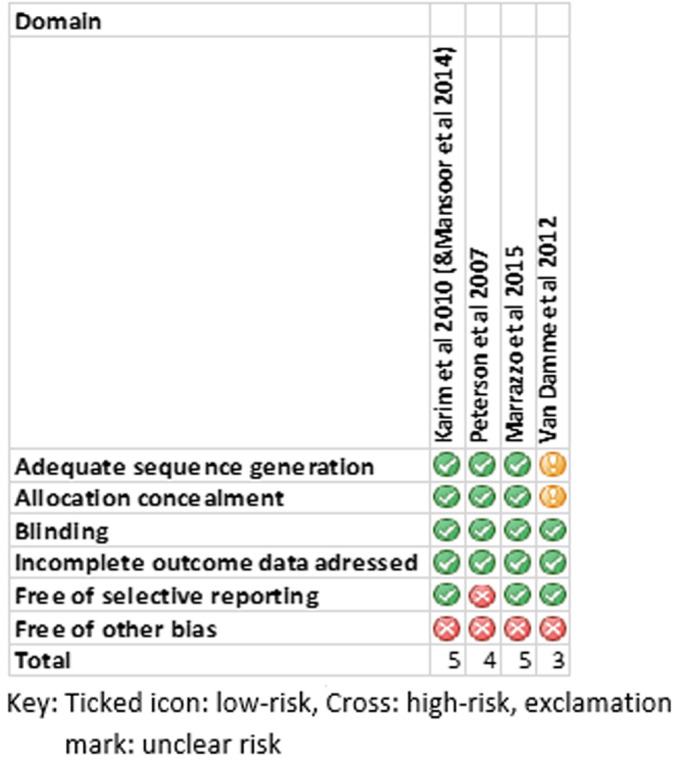
risk of bias assessments across included studies

**Risk of bias assessment:** Figure 2 shows the risk of bias across the four RCTs. Overall, the risk of bias was low; however, there were some sources of bias. Karim *et al*. [17], Peterson *et al*. and Marrazzo *et al*. [19] adequately described or referred to the study protocol for details regarding sequence generation, allocation concealment and blinding whereas van Damme *et al*. [18] did not. Thus, risk of bias was unclear for sequence generation and allocation concealment and double-blinding was concluded from the abstract and text. Peterson *et al*. selectively reported laboratory outcomes, excluding some from the Nigerian site [16]. Participants were not uniformly represented in all trials, with some sites overrepresented. However, due to effective randomisation noted this was probably inconsequential. Three of the studies [16,18,19] had all or part of them stopped prematurely, possibly failing to adequately power the studies.

**Synthesis:** the summary data for effectiveness of TFV-1% vaginal gel, oral TDF and TDF-FTC against their respective placebos are summarised in the table below. Owing to paucity of studies, we could not pool effect size estimates in meta-analyses and we synthesized the evidence qualitatively. The summary data are shown in Table 2. Karim *et al*. [17] and Marrazzo *et al*. [19] evaluated the effectiveness of TFV-1% vaginal gel. Two thousand eight hundred and ninety nine women contributing 3395.3 women-years of follow-up across the intervention and placebo arms. While Karim *et al*. reported an adjusted 37% protective effect of the gel against HIV acquisition that was statistically significant [17], Marrazzo *et al*. reported an insignificant 15% protective effect with a wide confidence interval [19]. When stratified according to gel adherence by Karim *et al*. [17], in high adherers (gel adherence >80%), HIV incidence was 54% lower (P=0.025) in the TFV-1% vaginal gel arm. In intermediate adherers (gel adherence 50 to 80%) and low adherers (gel adherence <50%), the HIV incidence reduction was 38 and 28%, respectively [17]. In a random sample in the study by Marrazzo *et al*. TFV was detected 25% of available plasma samples from participants assigned to TFV gel [19]. Detection of TFV in plasma was negatively associated with characteristics predictive of HIV-1 acquisition [19].

**Table 2:** summary data for effectiveness of TFV-1% vaginal gel, oral TDF and TDF-FTC against placebos

Product	Study	HIV endpoints/ no of participants	HIV incidence/100 women-years (95% CI)	HIV incidence rate ratio (±95% CI)	Adjusted hazard ratio (95% CI)	Effectiveness (95% CI)	Risk of bias
TFV gel	Karim *et al*. Science 2010	TFV gel arm: 38/445; placebo arm: 60/444	TFV gel arm, 5.6 (4.0-7.7); placebo arm, 9.1 (6.9-11.7)	0.61	0.63, (0.42-0.94); p=0.025	37% (6-58), statistically significant	Low
	Marrazzo *et al*. NEJM, 2015	TFV gel arm: 61/1007; placebo arm: 70/1003	TFV gel arm, 6.0 (4.6-7.6); placebo arm, 6.8 (5.3-8.6)	0.88	0.85 (0.61-1.21); p=0.37	15% (-21 to 39%), not statistically significant	Low
Oral TDF	Peterson *et al*. PLoS Clinical Trials, 2007	TDF arm: 2, 469; placebo arm: 6, 467	TDF arm, 0.86; placebo arm, 2.48	0.35 (0.03-1.93); p=0.24	-	65% (-93, 97%), statistically not significant	Medium
	Marrazzo *et al*. NEJM, 2015	TDF arm: 52, 1007; placebo arm: 35, 1009	TDF arm, 6.3 (4.7-8.3); placebo arm, 4.2 (2.9-5.8)	1.50	1.49 (0.97-2.29); p=0.07	49% increased risk, not statistically significant	Low
TDF-FTC	Van Damme *et al*. NEJM, 2012	TDF-FTC arm: 33, 1024; placebo arm: 35, 1032	TDF-FTC arm: 4.7; placebo arm: 5.0	0.94	0.95 (0.59-1.54); p=0.84	5% (-54 to 41%), not statistically significant	Medium
	Marrazzo *et al*. NEJM, 2015	TDF-FTC arm: 61, 1003; placebo arm: 60, 1009	TDF-FTC arm, 4.7 (3.6-6.1); placebo arm, 4.6 (3.5-5.9)	1.02	1.04 (0.73-1.49); p=0.81	4% increased risk (-49 to 27%), not statistically significant	Low

Peterson *et al*. [16] and Marrazzo *et al*. [19] evaluated the effectiveness of oral TDF against placebo. Two thousand five hundred and ninety two women were enrolled in the two studies combined, contributing 2134.9 women years of follow-up. None observed statistically significant protection. Marrazzo *et al*. terminated prematurely due to lack of effectiveness [19]. The study by Peterson *et al*. failed to reach the intended sample size and follow-up and thus was underpowered [16]. Peterson *et al*. did not provide HRs [16] whilst Marrazzo *et al*. did not provide IRRs [19]. Evaluation of effectiveness by Peterson *et al*. was limited by the low number of endpoints observed [16]. In a random sample, TFV was detected in 30% of available plasma samples from participants randomly assigned to receive TDF by Marrazzo *et al*. [19]. However, Peterson *et al*. did not provide any measure of adherence [16].

Marrazzo *et al*. [19] and van Damme *et al*. [18] evaluated the effectiveness of oral TDF-FTC against oral placebo. Four thousand one hundred and twelve women were enrolled across the arms in the two trials. No statistically significant protection was conferred. There was no notable clinical heterogeneity between the two studies. For all the interventions, only 2 studies were obtained, each from the 5-arm Marazzo *et al*. [19] RCT. Meta-analyses could thus not be conducted owing to this paucity of studies, thus we cannot provide pooled estimates for effectiveness of any of the study products. In a random sample by Marrazzo *et al*. TFV was detected in 29% of available plasma samples from participants randomly assigned to receive TDF-FTC [19]. In the study by van Damme *et al*. less than 40% of the HIV-uninfected women in the TDF-FTC group had evidence of recent pill use at visits that were matched to the HIV-infection window for women with sero-conversion [18].

## Discussion

This systematic review involving the synthesis of evidence from four studies that evaluated the effectiveness of TFV-based PrEP is one of the few reviews that have been conducted with emphasis on the use of PrEP prophylaxis to reduce transmission among SSA African women. Given that this population has unique socio-cultural and economic dynamics that place them at a higher risk of HIV acquisition [6,22-24], studies exploring effective prevention methods form the crux of reducing their HIV burden [25]. Though our synthesis suggests that TFV might not be effective in pre-exposure prophylaxis of HIV in this unique population, we critically appraised the evidence and unveiled poor adherence as a caveat that may guide researchers intending to conduct future studies in this or related domains.

Effectiveness was defined as the ability to prevent HIV infections in women on study products versus placebos and reported as IRRs or HRs. The IRR gave the relative differences in the HIV incidence rates in the active versus placebo arms whilst the HRs obtained from Cox regression models denoted the hazard of acquiring the infection for participants on active products over those on the placebo. We examined the effectiveness of TFV-1% vaginal gel, oral TDF and TDF-FTC against placebo but not against each other. Comparing them against each other and against placebo is called a network meta-analysis [26] and it useful for health technology assessment and economic evaluations; however, this requires significant expertise. None of the study products conferred statistically significant protection against HIV acquisition. The design, conduct and trial characteristics were similar though the dosing strategy for the gel was different [17-19]. The RCTs reviewed were well conducted, with low to medium risk of bias. They enrolled the appropriate group of SSA women at risk, aged 18-45 years. However, they were affected by premature stoppages resulting in inadequate follow-up or sample size, thus losing statistical power [16,18,19]. Low statistical power reduces chances of detecting the true effect but also reduces chances that a statistically significant effect represents a true effect. Thus, attaining adequate power is critical for high quality RCTs.

Jiang *et al*. conducted a meta-analysis of all TFV-PrEP (gel, TDF and TDF-FTC) against placebo and obtained 47% effectiveness (RR 0.53, 95% CI 0.40-0.71) [27]. This improved to 51% effectiveness (RR 0.49, 95% CI 0.38-0.63) in sensitivity analysis after excluding two studies with non-significant results. Seven RCTs were reviewed but Marrazzo *et al*. [19] was not included. Heterogeneous populations were included (TGW MSM, intravenous drug users (IDUs) and heterosexual men and women). Owing to differential risk perceptions, sexual behaviours, socio-economic characteristics it may not be appropriate to pool evidence from diverse key populations. Moreover, this meta-analysis was inappropriate because they conducted a pairwise meta-analysis where they combined the three interventions against a placebo. These three are unlikely to be equally efficacious and therefore should not be pooled together. Instead, a network meta-analysis may have been more appropriate [26].

Similarly, Okwundu *et al*. found PrEP to be effective at reducing risk of HIV infection in a Cochrane systematic review, with a statistically significant 51% reduction (RR 0.49, 95% CI 0.28-0.85) for TDF-FTC versus placebo and a statistically significant 67% reduction (RR 0.33, 95% CI 0.22-0.55) for TDF versus placebo [28]. The review included MSM, TGW, IDUs and heterosexual men and women across the world. The trial by Marrazzo *et al*. [19] was not yet reported. When considering the effectiveness across SSA women, these results may not be generalizable, thus the need for this review which was specific. Some of the studies we reviewed were stopped prematurely due to lack of efficacy [18,19]. While TFV-PrEP was protective of HIV acquisition in other populations, poor adherence was the caveat in the RCTs evaluating the effectiveness in women residing in SSA.

Objective measures of adherence in TFV-PrEP studies conducted in at-risk women residing in SSA consistently revealed low adherence, discordant from subjective reports [29-33]. Low adherence was noted in MTN-020, a study that evaluated the effectiveness (27%, 95% CI 1-46%) of a dapivirine vaginal ring against placebo in the same population [34]. When stratified according to adherence levels, the protective effect (37%, 95% CI 12-56%) of dapivirine increased by 10% in high-adherers [34]. That higher adherence is associated with a higher protective effect is also supported by evidence from Karim *et al*. and Marrazzo *et al*. [17,19]. A drug works best if it is in the right place, at the right time and in the right concentration. Qualitative studies among MSM reflect higher risk perception and commitment to PrEP, translating into higher adherence and effectiveness [35]. Detected blood levels of TDF-FTC strongly correlated with protective effect in the iPrEx trial [36,37]. As research advances towards multi-purpose prevention technologies for HIV, STIs and pregnancy, understanding the reasons why adherence remains low in this key at-risk population is critical, as the progress and success of future clinical trials premises upon addressing the inadequacies of adherence.

**Strengths:** this systematic review was conducted and reported using the PRISMA-2009 guidelines [10], following a protocol that had been developed according to PRISMA-P 2015 guidelines [11]. This is now considered the gold standard for conducting and reporting systematic reviews. Multiple databases were searched to maximise chances of retrieving all eligible studies and minimise bias as per Cochrane recommendations [12]; registries of trials were searched to note completed and ongoing trials. The literature search was progressively built from an initial scoping search in Medline, from which the comprehensive final search was refined. Sensitivity-maximising filters from the Cochrane collaboration and SIGN were utilised to identify all eligible studies. There was no language restriction to eliminate language bias, though all the eligible studies were in English, negating the need for translation. By sticking to PRISMA and Cochrane guidelines, this review was meant to be replicable.

The studies included in this review were all carried out among SSA women, absolute age ranges 18-45 years, the group at highest risk of HIV acquisition, with comparable demographic and socio-economic characteristics. The multi-centre, multi-national RCTs extend external validity to comparable populations across SSA.

**Limitations:** a limited number of studies were retrieved. Thus, publication bias and statistical heterogeneity could not be explored. Meta-analyses, funnel plots, meta-regression and cumulative meta-analysis, which allow further explorations, as well as sensitivity analyses, could not be conducted. We could not provide pooled effect size estimates of interventions owing to paucity of studies.

**Implications for public health and research:** the burden of HIV among young SSA women remains substantial. Preventive public health interventions are the key to curtailing further infections and whilst the search for an effective vaccine continues, we must investigate other effective interventions in addition to the array that already exists. TVF-PrEP offers such an attractive choice for women. Whilst in September 2015 the WHO recommended the daily use of TFV-PrEP in at-risk groups such as MSM, TGW and heterosexual men and women in sero-discordant couples [4], the evidence to extend this intervention to SSA women remains missing. Whilst exploring further the reasons for effectiveness, the search for other effective interventions to protect SSA women from HIV acquisition must continue. As science advances towards multipurpose prevention technologies for women, barriers towards uptake of protective interventions that work in other populations must be addressed among SSA women.

## Conclusion

None of the study products, TFV-1% vaginal gel, oral TDF and TDF-FTC conferred statistically significant protection against HIV acquisition in young at-risk SSA women. The current evidence does not support the effectiveness of TFV in the pre-exposure prophylaxis for HIV among women residing in SSA. Due to the paucity of studies, more studies are needed and factors that may affect effectiveness such as adherence need to be further explored.

### What is known about this topic

- Tenofovir-based pre-exposure prophylaxis is effective for preventing HIV infections among men who-have-sex with men and transgender women;
- Effectiveness of tenofovir-based pre-exposure prophylaxis correlates with objectively measured drug levels in clinical trials.

### What this study adds

- Uniquely, among sub-Saharan African women, tenofovir-based pre-exposure prophylaxis was not effective in preventing new HIV infections;
- Adherence was the missing link between efficacy and effectiveness of tenofovir-based pre-exposure prophylaxis for preventing new HIV infections among sub-Saharan African women, necessitating the need to explore further the factors leading to low adherence to effectively inform future studies.

## Annexes

**Annex 1**: OVID MEDLINE search strategy (PDF - 313Kb)

**Annex 2**: OVID embase search strategy (PDF - 317Kb)
